# Structure of Water in the First Hydration Shell of the Highly Hydrophobic Anthracene

**DOI:** 10.1021/acs.jpca.6c02910

**Published:** 2026-08-03

**Authors:** Gustavo A. Madrigal, Christos Vasilopanagos, Thomas F. Headen, Alexander Rosu-Finsen, Bharvi Chikani, Sukhpreet K. Talewar, Luis Carlos Pardo, Christoph G. Salzmann

**Affiliations:** † Barcelona Research Center in Multiscale Science and Engineering, Universitat Politècnica de Catalunya, 08019 Barcelona, Spain; ‡ Department of Physics, Universitat Politècnica de Catalunya, 08019 Barcelona, Spain; § 4919 Department of Chemistry, University College London, 20 Gordon Street, London WC1H 0AJ, U.K.; ∥ ISIS Pulsed Neutron and Muon Source, STFC Rutherford Appleton Laboratory, Harwell Campus, Harwell OX11 0QX, U.K.

## Abstract

Hydrophobic hydration underpins processes central to chemistry, biology, and technology, from protein folding and molecular self-assembly to catalysis and nanomaterial dispersion. However, direct structural information on water surrounding large hydrophobic molecules remains scarce due to their very low solubility. Here, we overcome this limitation by using low-temperature vapor codeposition to form amorphous mixtures of water and anthracene, a hydrophobic “mini-graphene”. We then resolve the structure of anthracene’s first hydration shell by neutron diffraction. The linear polycyclic aromatic hydrocarbon induces distinct structural motifs in its hydration shell. Water molecules above and below the outer aromatic rings exhibit one broken water–water hydrogen bond enabling weak O–H···π interactions with the solute, while a pronounced “dewetting” effect emerges at the central ring. These findings provide experimentally constrained structural insights into the hydration around a large hydrophobe under conditions where conventional liquid-state experiments are not feasible.

## Graphical abstract

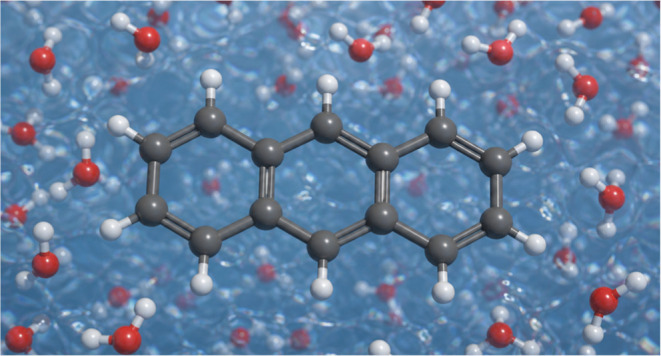

## Introduction

Hydrophobic hydration is the structural and thermodynamic response of water molecules to the presence of nonpolar, water-repelling molecules or surfaces.^1–4^ Many processes central to life are driven by phenomena associated with hydrophobic hydration, particularly in biomolecular self-assembly such as protein folding, and the formation of micelles and membranes.^5–7^ It also underpins a wide range of disease-related processes including the formation and growth of amyloid fibrils.^8,9^ Beyond biology, hydrophobic hydration plays a key role in numerous technological and industrial applications, ranging from the industrial treatment of minerals^10^ and chemical catalysis,^11,12^ to the promotion of clathrate-hydrate growth,^13–15^ and the dispersion of hydrophobic nanomaterials in water.^16^

The size of a hydrophobic molecule has a significant impact on how it is hydrated. For small hydrophobic solutes in water, such as methane or other small hydrocarbons, the hydration process is governed by an unfavorable entropy loss.^4^ Water molecules arrange themselves in more ordered, lower entropy structures around the solute compared to the bulk liquid thereby reducing their freedom of motion. The ordered structure of such hydration shells was first described by Frank and Evans in terms of the iceberg model.^1^ The suggestion is that the water molecules form hydrogen-bonded cages around small hydrophobes similar to the ones found in the crystalline clathrate hydrates.^17,18^ In addition to structural effects, decreases in the mobilities of the water molecules in the hydration shells of small hydrophobes contribute to the entropy loss.^19,20^

For larger hydrophobic solutes and extended surfaces, the hydration is thought to be dominated by enthalpic effects as it becomes necessary, due to geometric constraints, to reduce the number of hydrogen bonds between the water molecules.^4^ Consequently, the water molecules tend to minimize hydrophobic contacts which drives phenomena such as the aggregation of hydrophobic surfaces, and the assembly of micelles or membranes. Surface-drying effects akin to the formation of liquid–vapor interfaces as a consequence of the hydrophobic hydration were first envisaged by Stillinger^2^ and this phenomenon is of particular relevance in the context of hydrophobic pockets.^21^

The changeover between the two different regimes of hydrophobic hydration has been termed the hydrophobic crossover by Lum, Chandler and Weeks.^3,4,22^ Although not yet observed experimentally for truly hydrophobic species, the hydrophobic crossover has been estimated computationally to occur at solute diameters around 10 Å.^23^ The fundamental problem in experimentally reaching the hydrophobic crossover is that truly hydrophobic molecules become increasingly less soluble in water as their size increases which in turn means that the hydration water becomes progressively more challenging to investigate. Experimental studies attempting to pin down the hydrophobic crossover have therefore so far relied on using amphiphilic molecules where the hydrophilic part of a molecule enables its solubility. The experimental techniques employed so far have included vibrational spectroscopy,^24–26^ measurements of thermodynamic quantities^27,28^ and neutron diffraction.^29^ The characteristic length scale of the hydrophobic crossover is expected to increase with decreasing temperature highlighting the delicate competition between entropic and enthalpic contributions.^23,30,31^

In computer simulations, the description of hydrophobic hydration remains challenging, as it requires highly accurate water models, sufficiently large simulation boxes, and a detailed treatment of the time dependence of the dynamic processes. The balance between the competing entropic and enthalpic terms has been explored using molecular dynamics simulations.^32,33^ Modern theoretical and simulation studies have established that hydrophobic hydration is inherently length-scale dependent, with a gradual crossover from small hydrophobes, which only weakly perturb the surrounding hydrogen-bond network, to larger hydrophobic surfaces, where interfacial effects and partial dewetting become increasingly important.^22,34,35^ This refined picture has largely replaced a literal interpretation of the Frank–Evans iceberg model. While small hydrophobic solutes can induce subtle increases in local tetrahedral ordering, these effects are modest and temperature dependent rather than corresponding to rigid ice-like shells.^36–38^ Nevertheless, some recent molecular dynamics studies have argued that transient, locally ordered water aggregates consistent with a moderate interpretation of the Frank–Evans iceberg concept can emerge around hydrophobic solutes, particularly at lower temperatures.^39^ For larger hydrophobic surfaces, the hydration layer is better described as a fluctuating liquid–vapor-like interface with enhanced density fluctuations.^40,41^ The aggregation force between two hydrophobic plates has been shown to depend on the size of the plates and the exact details of the water–plate interactions,^42^ while simulations of planar hydrophobic surfaces indicate that orientational ordering of the hydration layer precedes the onset of pronounced dewetting.^43^ Drying phenomena at larger hydrophobic surfaces have been described in terms of near-critical phenomena.^44^ This framework has then been applied to understanding the temperature-dependent aggregation forces between two hydrophobic spheres.^31^ Given the complexity of treating hydrophobic hydration computationally, the benefits of using coarse-grain approaches have been outlined.^45^ Clearly, further experimental studies are sought-after as they will help develop and benchmark computational approaches.

To overcome the experimental obstacle posed by the very low solubilities of larger hydrophobic molecules, we recently developed an experimental technique for the codeposition of the vapors of water and a hydrophobe onto a cryogenic surface at 80 K.^46^ Using this method, we successfully achieved the matrix isolation of the hydrophobic hydrocarbon adamantane (C_10_H_16_) in amorphous ice and investigated the structure of its first hydration shell with neutron diffraction in combination with the isotopic substitution method.^47^ The almost spherical adamantane molecule has a van der Waals diameter of 6.8 Å and we found that its first hydration shell resembles a 5^12^6^4^ cage of fully hydrogen-bonded water molecules with 12 pentagonal and four hexagonal faces, as it is also found in cubic structure II clathrate hydrates where this cage type combines with 5^12^ cages.^47^ The fact that water molecules form fully hydrogen bonded 5^12^6^4^ cages around adamantane means that adamantane is still too small a hydrophobe to induce the structural changes associated with the hydrophobic crossover. Evidently, to experimentally observe hydration structures beyond the hydrophobic crossover, the size of the hydrophobic solute needs to be increased further beyond the size of adamantane. It should be stressed that our structural characterizations were carried out at 80 K. Any relationship to the hydrophobic crossover, which has predominantly been investigated for liquid water near room temperature, therefore requires careful discussion. Interestingly, our previously reported hydration structure of adamantane at 80 K^47^ is fully consistent with the original Frank–Evans iceberg model.^1^

In this work, we mix water and the hydrophobic aromatic hydrocarbon anthracene (C_14_H_10_). The solubility of anthracene in liquid water at room temperature is extremely low at around 3 × 10^–7^ mol L^–1^.^48^ This on the one hand illustrates its highly hydrophobic nature and on the other hand the necessity of our low-temperature approach to “dissolve” anthracene in water. Anthracene is a polycyclic aromatic hydrocarbon (PAH) consisting of a linear array of three fused benzene rings and it has a van der Waals diameter of 13 Å measured along its long axis. In a sense, anthracene can be regarded as a “mini-graphene” and therefore a nanoscale hydrophobic surface. Its length exceeds the hydrogen-bonded distance between water molecules four times, which makes it a promising candidate for pushing beyond the hydrophobic crossover. As successfully employed for hydrated adamantane,^47^ neutron diffraction in combination with the isotopic substitution method was used to gain experimental insights into the structure of the first hydration shell of the hydrophobic anthracene.

## Experimental and Computational Methods

### Materials

Protiated (C_14_H_10_) and deuterated (C_14_D_10_) anthracene were obtained from Sigma-Aldrich with purities of ≥99% and 98 at % D, respectively. Ultrapure water (H_2_O, Milli-Q, Millipore) and heavy water (D_2_O, 99 at % D) were also purchased from Sigma-Aldrich.

### Preparation of Amorphous Mixtures of Water and Anthracene

The experimental setup for the cryogenic matrix isolation of anthracene in amorphous ice was based on a high-vacuum box chamber purchased from Kurt Lesker as shown schematically in Figure 1a. This setup had previously been used to prepare amorphous mixtures of water/C_60_ fullerene^46^ and water/adamantane.^47^ It features a top-loading system for liquid nitrogen to cool an 8-in. diameter copper deposition plate. A removable cold trap, positioned before the diffusion pump, facilitates easy cleaning of anthracene residues.

**Figure 1:**
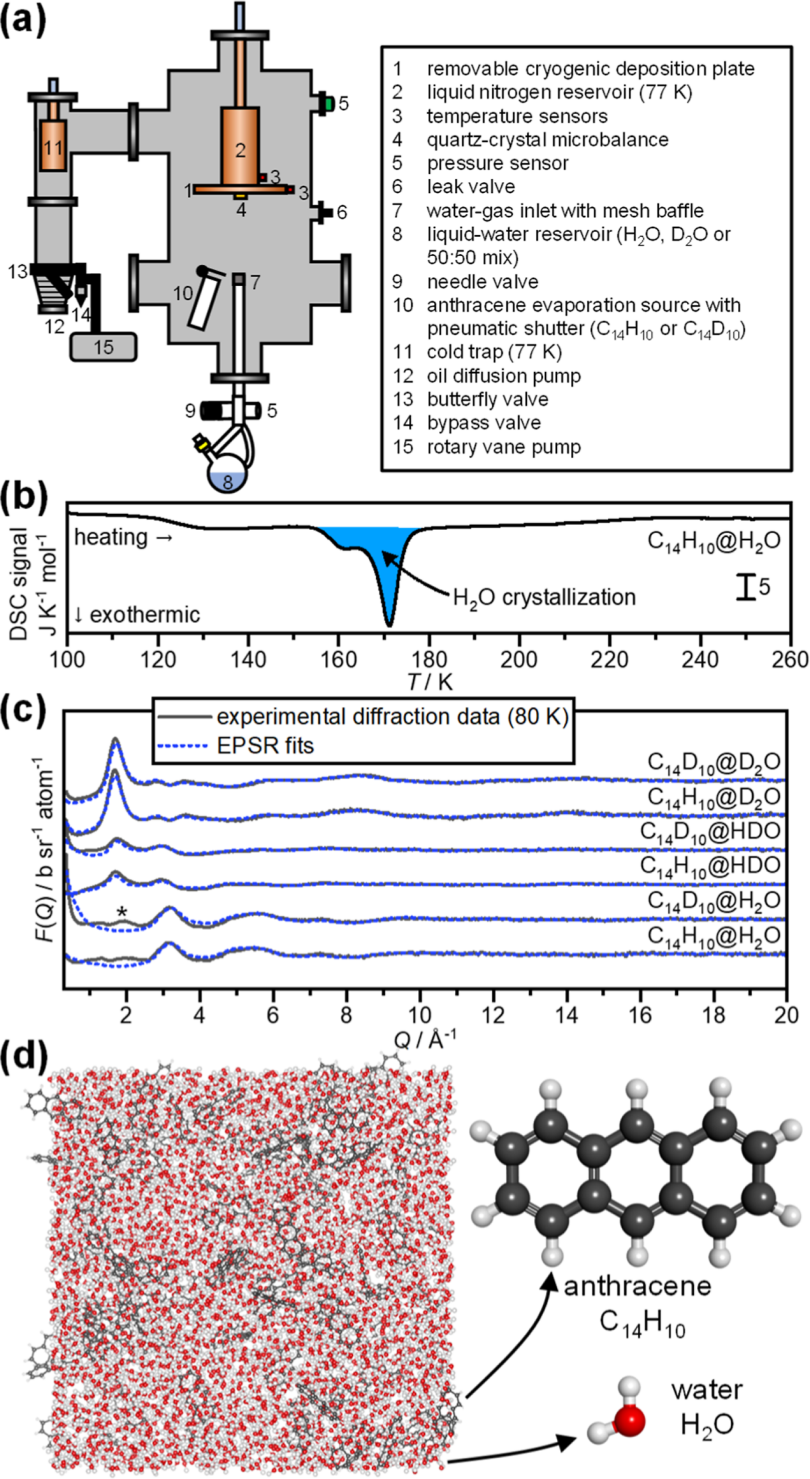
Preparation of amorphous mixtures of anthracene (C_14_H_10_) and water, and analysis of the neutron diffraction data. (a) Schematic illustration of the vacuum-chamber setup used for the vapor codeposition of anthracene and water onto a cold substrate. The various components are numbered and listed in the legend. (b) Differential scanning calorimetry data recorded upon heating C_14_H_10_@H_2_O (1:45) (c) Experimental neutron diffraction data set of six amorphous mixtures of anthracene and water with molar ratios of 1:45 and different protium/deuterium contents as indicated (black lines). Empirical Potential Structure Refinement (*EPSR*) fits to the experimental diffraction data are shown as blue dotted lines. The asterisk marks a diffraction feature due to an unknown impurity. (d) *EPSR* simulation box consistent with the experimental neutron diffraction data set and the molecular structure of anthracene. The carbon, oxygen and hydrogen atoms are shown as black, red and white spheres.

The temperatures of both the deposition plate and the liquid nitrogen reservoir were monitored using K-type thermocouples connected to a custom-built instrument comprising an Adafruit Feather 32u4 microcontroller and two Adafruit MAX31856 Universal Thermocouple Amplifiers. The pressure in the chamber was measured with a combined Pirani/cold cathode gauge (PenningVac PTF 90, Oerlikon Leybold Vacuum) which typically reached 2 × 10^–6^ mbar.

A 32 cm long, 2.6 cm diameter stainless-steel tube at the bottom of the chamber allowed water vapor to be introduced toward the deposition plate, which was positioned 15 cm above the inlet. The water-inlet system included a needle valve (EV 016 DOS AB, Oerlikon Leybold Vacuum) to regulate water-vapor flow and a Pirani pressure sensor (Thermovac TTR91, Oerlikon Leybold Vacuum) to monitor the inlet pressure. Liquid water was held in a round-bottom flask fitted with a Young’s tap connected to the inlet system. Before introducing H_2_O, D_2_O, or a 50 mol% mixture into the chamber, several freeze–pump–thaw cycles were performed to degas the water. To diffuse the water-vapor flow, an iron mesh with 0.5 mm thick wires spaced 1 mm apart was placed directly above the water inlet tube.

The vaporization of anthracene was achieved using an LTE-10 point-source evaporator from Kurt Lesker equipped with a pneumatically operated shutter. For each run, 1.5 g of anthracene powder was loaded into the 15 cm^3^ Al_2_O_3_ crucible of the evaporator and compacted using a piston. The source temperature was monitored via a K-type thermocouple and controlled by a Eurotherm 2408 PID unit integrated into a MAPS power supply (Kurt Lesker).

At the start of a deposition experiment, the chamber was evacuated to a base pressure of 2 × 10^–6^ mbar and the deposition plate was cooled to 82 K. Water vapor was introduced by adjusting the needle valve to achieve an inlet pressure of 1.00 × 10^–1^ mbar. The anthracene evaporation source was heated with the shutter closed and, once the target temperature was reached, the shutter and needle valve were simultaneously opened to begin the codeposition.

The deposition rates of both water and anthracene were calibrated using a quartz-crystal microbalance (QCM) comprising gold-plated AT-cut 6 MHz plano-convex quartz crystals (Inficon) mounted in an Allectra 710-SH sensor at the center of the deposition plate. This was connected via coaxial cables to a reflection bridge and a 0.5–60 MHz N2PK vector network analyzer. Changes in the crystal’s fundamental frequency were tracked during deposition using the myVNA and QTZ software packages,^49^ and the deposition rates were calculated by applying a linear fit to the frequency-versus-time data.

For H_2_O deposition, an inlet pressure of 1.00 × 10^–1^ mbar yielded a deposition rate of 0.0363 ± 0.0001 μmol cm^–2^ s^–1^. The deposition rates of D_2_O and the 50 mol%H_2_O/D_2_O mixture were equivalent within experimental error indicating ideal-gas behavior under these conditions. Rapid isotopic exchange in the liquid H_2_O/D_2_O mixtures led to a statistical distribution of water isotopologues. For an overall H/D ratio of 1:1, the mixture is therefore expected to contain approximately 50% HDO, 25%H_2_O and 25% D_2_O molecules. For simplicity, these samples are labeled as HDO mixtures.

The deposition rates of C_14_H_10_ and C_14_D_10_ were calibrated by varying the evaporation source temperature between 50 and 100 °C. To achieve a 1:45 molar ratio of anthracene to water, C_14_H_10_ and C_14_D_10_ were maintained at 71 and 78 °C, respectively. In total, six amorphous 1:45 mixtures were prepared with the following isotopic compositions: C_14_H_10_@H_2_O, C_14_H_10_@HDO, C_14_H_10_@D_2_O, C_14_D_10_@H_2_O, C_14_D_10_@HDO and C_14_D_10_@D_2_O.

To stop the deposition, the pneumatic shutter and the needle valve were closed. The samples were then annealed by heating to 125 K under vacuum to close the pores of the amorphous deposits. After cooling back to base temperature, the vacuum chamber was leaked to dry and cold nitrogen gas. The deposition plate was then quickly removed from the chamber under liquid nitrogen. The samples were scraped off with a decorator knife and transferred into flat-plate TiZr cans. Typical deposition times were around 4 h yielding approximately 3 g of material.

### Neutron Diffraction Experiment and Data Normalization

The internal dimensions of the null-scattering Ti_0.68_Zr_0.32_ alloy sample cells were 1 × 38 × 38 mm for the H_2_O samples and HDO with C_14_H_10_, and 2 × 38 × 38 mm for the D_2_O samples and HDO with C_14_D_10_. Neutron scattering measurements were conducted at 80 K on the NIMROD (Near and Intermediate Range Order Diffractometer) diffractometer at the ISIS spallation neutron source, Rutherford Appleton Laboratory, UK, using a proton beam current of 30 μA, with each sample measured to an integrated proton current of 150 μA h.^50^ NIMROD collects neutrons scattered at angles between 0.5 and 40° covering a wavevector transfer range of 0.02–50 Å^–1^.^50^

Raw scattering data were corrected for absorption and multiple scattering using the *GudrunN* software package,^51^ which also accounts for perturbations from inelastic collisions in particular with protium. The “Iterate Gudrun” feature was employed to further eliminate inelasticity effects and obtain the total structure factors, *F*(*Q*), of the samples.^52^ These corrections were performed using the iterative subtraction of wavelength-binned data employing a flat background subtraction determined from the average intensity above *Q* = 10 Å^–1^. Five iterations were performed, resulting in convergence of the corrected *F*(*Q*).

### Fitting the Neutron Diffraction Data Set

The Empirical Potential Structure Refinement (*EPSR*) technique^53^ within the *Dissolve* software^54^ was used to fit the experimental diffraction data set and generate a structural model of the samples. Detailed descriptions of the neutron scattering theory and the *EPSR* approach are given in the Supporting Information of refs 55 and 56. *Dissolve* begins with a full classical force field Monte Carlo (MC) simulation employing standard reference potentials to describe both inter- and intramolecular interactions. To ensure that the intramolecular structure of the molecules remains consistent with the full force field, short molecular dynamics (MD) simulations of 50 steps with a time step of 0.5 fs are performed every five MC iterations. Unlike the MC moves, which consist of whole-molecule translations and rotations, the MD component operates on a per-atom basis and therefore maintains the molecular geometry dictated by the force field. From the evolving simulation, the predicted scattering patterns for each isotopologue are calculated and compared with the experimental data. The discrepancies are used to iteratively construct an additional empirical potential, which is added to the reference potentials to progressively refine the simulation and achieve agreement across the entire data set. The contributions of the various partial structure factors to the total structure factors are given in Table S1.

Each simulation was conducted in a cubic box with an edge length of 55.3 Å, containing 4500 water and 100 anthracene molecules, corresponding to a number density of 0.0942 atoms Å^–3^. Molecular parameters for anthracene, including average bond lengths and angles, 12–6 Lennard-Jones potentials and partial charges, were taken from the OPLS-AA (Optimized Potentials for Liquid Simulations – All Atoms) force field.^57^ The SPC/E water model was used to describe water molecules.^58^ The details of the seed potentials are given in Table S2. While the seed potentials used in the *EPSR* structural refinement are classical, there is no *a priori* reason why the structural refinement should not capture complex intermolecular structures required by the experimental neutron scattering data. We note that the high symmetry of anthracene is advantageous for obtaining robust structural refinements as it keeps the number of partial structures factors small. After convergence of the *EPSR* fits, approximately 20,000 configurations were accumulated.

### Structural Analyses

The *ANGULA* software^59^ was used to analyze the configurations including the locations of the oxygen atoms and orientations of the water molecules in the first hydration shell of anthracene as well as the structures of their hydrogen-bonded environments.

### Differential Scanning Calorimetry

After removal from the vacuum chamber, the samples were transferred into stainless-steel DSC pans and rapidly loaded into a precooled PerkinElmer DSC 8000 Advanced Double-Furnace Differential Scanning Calorimeter. The samples were heated from 93 to 263 K at a rate of 10 K min^–1^, resulting in the formation of bulk ice I*h* and anthracene. A second heating scan was subsequently recorded and used for background subtraction. Finally, the ice was melted at 0 °C. The number of moles of H_2_O was determined from the enthalpy of melting using a molar enthalpy of fusion of 6012 J mol^–1^. The background-corrected DSC signal was then normalized by dividing by the number of moles of H_2_O and the heating rate, yielding a quantity in units of J mol^–1^ K^–1^.

### X-ray Diffraction

The recovered samples were transferred under liquid nitrogen into custom-made sample holders equipped with Kapton windows. For collecting X-ray diffraction patterns, the holders were then mounted on a Stoe Stadi-P X-ray transmission diffractometer (Cu Kα_1_ radiation, 40 kV, 30 mA) fitted with a Ge(111) monochromator and a Mythen 1K linear detector. The sample temperature was initially held at 95 K and subsequently increased from 100 to 270 K in 10 K increments using a CryojetHT (Oxford Instruments).

## Results and Discussion

Mixtures of water and anthracene were prepared in a vacuum chamber designed for the simultaneous vapor deposition of both species onto a cold surface.^46,47^ A schematic of the setup is shown in Figure 1a. The main components include a removable deposition plate, which can be cooled to ∼82 K, a heated evaporation source for anthracene equipped with a pneumatic shutter, and a water-vapor inlet supplied from a liquid-water reservoir underneath the chamber. The deposition rates of both species were calibrated with a quartz-crystal microbalance (QCM) mounted on the deposition plate, and controlled by adjusting the evaporation-source temperature for anthracene and a high-precision needle valve for the water vapor.^46,47^

A target molar ratio of 1:45 (anthracene/water) was chosen to balance two competing requirements: minimizing the aggregation of adjacent anthracene molecules while maintaining sufficient scattering contributions from the anthracene–water interface. The amorphous nature of the deposits was verified by X-ray diffraction at 80 K, which revealed the broad diffraction features characteristic of amorphous solids (Figure S1). Upon heating, the amorphous samples displayed an exothermic phase transition at ∼ 155 K in calorimetry due to the crystallization of ice (Figure 1b). This was confirmed by variable-temperature X-ray diffraction where additionally the crystallization of anthracene could be seen starting sluggishly above ∼200 K (Figure S2).

Six amorphous anthracene–water samples with 1:45 molar ratios were then prepared using different combinations of deuterated and protiated anthracene and water. The neutron-diffraction patterns of these samples are shown in Figure 1c. The broad features again confirm the amorphous character of the deposits, demonstrating the successful mixing of anthracene and H_2_O. The diffraction data set was analyzed using the Empirical Potential Structure Refinement (*EPSR*) method, which iteratively optimizes a structural model to best reproduce the experimental diffraction data.^53^ Employing H/D isotopic contrasts enhances the reliability of the resulting structural models, one of which is shown in Figure 1d. After convergence of the fitting procedure, ∼20,000 structures were saved for detailed structural analyses of the amorphous mixtures. A comparison of the experimental and computed total pair distribution functions is shown in Figure S3.

Extracting insightful structural information from such highly disordered simulation boxes is inherently challenging, a difficulty further compounded by the markedly different van der Waals radii of anthracene along its principal axes. Hence, standard pair-distribution functions from the center of mass of the anisotropic anthracene to the oxygen atoms of water would fail to give meaningful structural information. To overcome this obstacle, we implemented a new search algorithm within our *ANGULA* software^59^ that identifies the shortest distances between the oxygen atoms of the water molecules and the nearest hydrogen or carbon atoms of the closest anthracene molecule. These distances, denoted *r*_O→(C/H)_, are illustrated schematically in Figure 2a. For comparison, the ‘traditional’ intermolecular anthracene to water pair distribution functions are shown in Figure S4.

**Figure 2:**
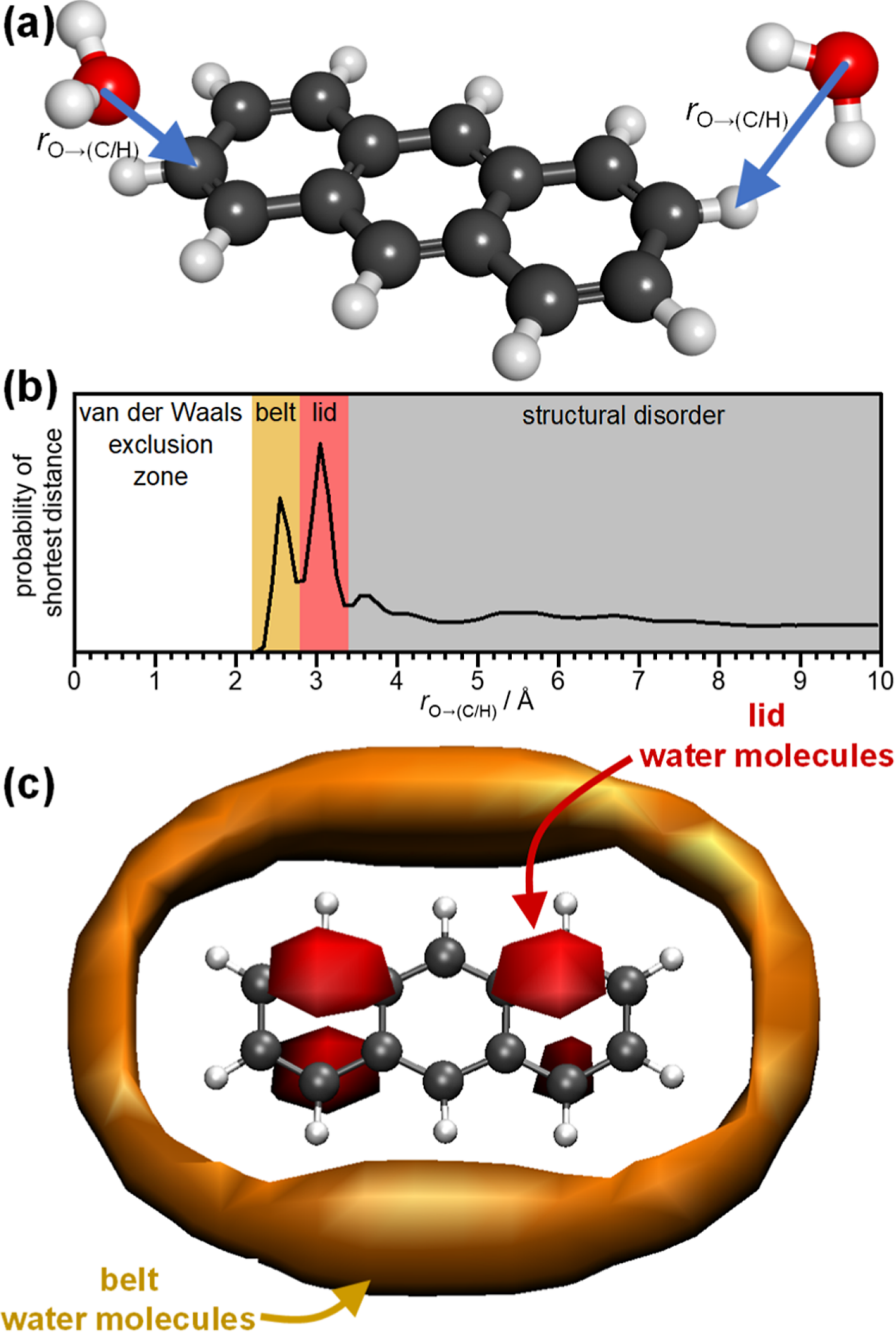
Locations of the oxygen atoms of water in the first hydration shell of anthracene. (a) Schematic illustration of the closest distances between the oxygen atoms of water molecules and either the carbon or hydrogen atoms of anthracene, *r*_O→(C/H)_. (b) Probabilities of finding the shortest *r*_O→(C/H)_ with increasing distances away from anthracene. (c) Volumes highlighting the most likely positions of the water oxygen atoms in the first hydration shell. The two different regions are labeled as belt (orange) and lid water molecules (red).

The probability function related to *r*_O→(C/H)_ in Figure 2b shows two distinct hydration regimes of anthracene with maxima located at 2.55 and 3.05 Å, respectively. At *r*_O→(C/H)_ distances beyond 3.4 Å, the structure is disordered indicating that only the first hydration shell of anthracene displays a significant degree of structural order.

The first hydration shell of anthracene exhibits two predominant water locations: around the “belt” of the flat molecule, and above or below its outer aromatic rings. These regions correspond to the two volumes highlighted in Figure 2c and fall within the distance ranges of the peaks observed in Figure 2b. Depending on the locations within the first hydration shell, we label the water molecules as either belt or lid molecules.

Strikingly, the probability of finding water directly above or below the central aromatic ring is very low. To quantify this effect, two spherical sectors were defined: one originating from the center of the central ring of anthracene and one from the center of an outer ring, with both sectors pointing away from the aromatic plane. Both spherical sectors had an opening angle of 126.9° [cos­(*θ*) = ± 0.6] and a radial cutoff of 3.6 Å, which is large enough to include the lid-type water molecules. Integration of the *ANGULA* angular distribution shows that the region between the two lid-like hydration populations accounts for only 3.8% of the population, confirming a strong depletion of water in this part of the closest hydration shell.

This depletion or “dewetting” reflects most likely a steric blocking effect, whereby water molecules more closely hydrating the outer aromatic rings restrict neighboring molecules from accessing sites near the central ring due to the directionalities of the hydrogen bonds. This appears to be a similar situation compared to the drying effects observed computationally at hydrophobic nanopockets which are governed by the hydrogen bonding of the water molecules at the rim.^21^

The orientations of the belt water molecules with respect to anthracene are similar to those observed in clathrate-hydrate type cages where the water molecules have four hydrogen-bonded neighbors. To analyze the orientations, two different angles were defined as shown in Figure 3a. First, a vector within the plane of anthracene and away from the center of the middle ring is defined. *θ*_O–H_ then describes the angle between this vector and one of the O–H bonds of the water molecule. The deflection of the dipole moment away from the vector is defined as *θ*_dp_. The probabilities of finding certain values of these two angles are shown in Figure 3b. The most likely orientations of the O–H bonds are either around 0° or the tetrahedral angle of 109.5°. Consequently, the dipole moments take values above and below 90° in line with orientational disorder of hydrogen-bonded water molecules.

**Figure 3:**
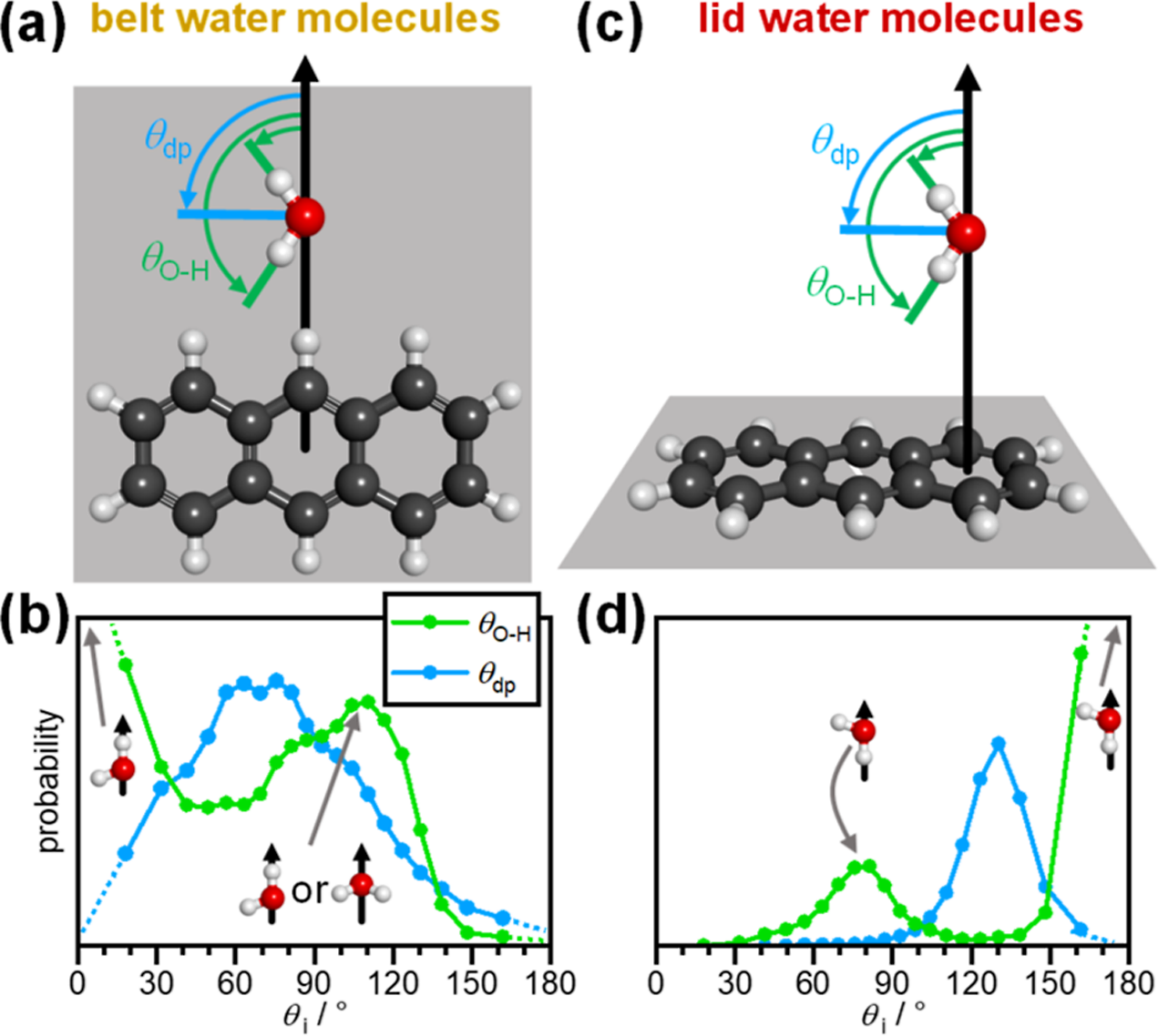
Orientations of the water molecules in the first hydration shell of anthracene. Definitions of the angles of the (a) covalent O–H bonds, *θ*_O–H_, and (b) dipole moments of water molecules, *θ*_dp_, with respect to the normal vector either from (a) the center-of-mass of anthracene to belt water molecules or (b) from the centers of the other six-membered rings of anthracene to lid water molecules. Probabilities of finding water molecules with the two different angles for (c) belt and (d) lid water molecules.

The lid water molecules on the other hand display a preference for one of the O–H bonds to point toward anthracene. For the angle analysis, a different vector is now defined from the center of the outer aromatic rings and perpendicular to the aromatic plane as shown in Figure 3c. The two peaks in the *θ*_O–H_ probability function are located around 70 and 180°. Consistent with one of the O–H bonds pointing toward the anthracene, *θ*_dp_ has a maximum at around 130°. This orientation suggests that the lid water molecules are only hydrogen-bonded to three neighboring water molecules. For such a configuration, there would still be the possibility for one of the oxygen electron lone pairs to point toward the anthracene. However, this does not seem to be realized in our sample suggesting the presence of noncovalent O–H···π interactions between water and anthracene.

Such O–H···π interactions have also been found experimentally for benzene/water both in the gas^60^ and in the liquid phase,^61^ and computationally for gas-phase clusters of anthracene^62^ or naphthalene^63^ with a few water molecules. The energetically most favorable geometry of a single water molecule on top of anthracene has the water molecule located above the central rings of anthracene with the two O–H bonds pointing toward the two outer rings.^62^ This geometry, which includes two O–H···π interactions, has a binding energy of 3.7 kcal mol^–1^. Upon increasing the number of water molecules around anthracene, clusters similar to those of pure water in the vapor phase are observed, which illustrates that hydrogen bonding between the water molecules is stronger than the O–H···π interactions. The strength of hydrogen bonding between water molecules is cooperative which means that hydrogen bonds become increasingly more difficult to break as the number of hydrogen bonds increases.^64^ The cohesive energy of ice has been estimated as 6.8 kcal mol^–1^.^65^ The fact that more energy is required to break the water–water O–H···O hydrogen bonds compared to the energy gained by O–H···π bonds is important since otherwise the hydration of anthracene would not be governed by hydrophobic hydration effects.

In addition to O–H···π bonding, weak C–H···O interactions have been discussed in the context of naphthalene–water clusters which may be relevant for the belt molecules in our sample.^63^ Figure S5a shows that the most likely distance between the hydrogen atoms of anthracene and the oxygen atoms of belt molecules is around 2.75 Å. Using a H···O cutoff distance of 3.0 Å, the C–H···O angles were found to deviate from linearity as can be seen in Figure S5b. The most frequently observed C–H···O angle was found at 149.6°. In this context it is worth mentioning that in a recent neutron diffraction study of liquid methanol–benzene mixtures at room temperature, methanol was found to solvate benzene through two structural motifs: methanol hydroxyl groups forming directional O–H···π hydrogen bonds above and below the aromatic ring, and methanol molecules lying approximately coplanar with the ring and interacting via bifurcated C–H···O hydrogen bonds.^66^ This closely resembles our belt-and-lid model for hydrated anthracene, with the main difference being that our solvation shell consists of a hydrogen-bonded network of water molecules rather than methanol chains.

A systematic analysis of the hydrogen-bonding environments of the lid water molecules confirms that they form indeed three hydrogen bonds with neighboring water molecules, while the remaining O–H group points toward the outer aromatic rings of anthracene. Figure 4a shows the running coordination number, *N*(*r*_O–O_), which quantifies the increase in coordination number as a function of the O–O distance away from a lid water molecule. As expected for water molecules with three hydrogen bonds, *N*(*r*_O–O_) plateaus at 3 in the 3.1–3.4 Å range for the lid molecules. This ‘inverted cup’ structure including three water–water hydrogen bonds and one O–H···π interaction is shown by the snapshot structure in Figure 4b and schematically in Figure 4c.

**Figure 4:**
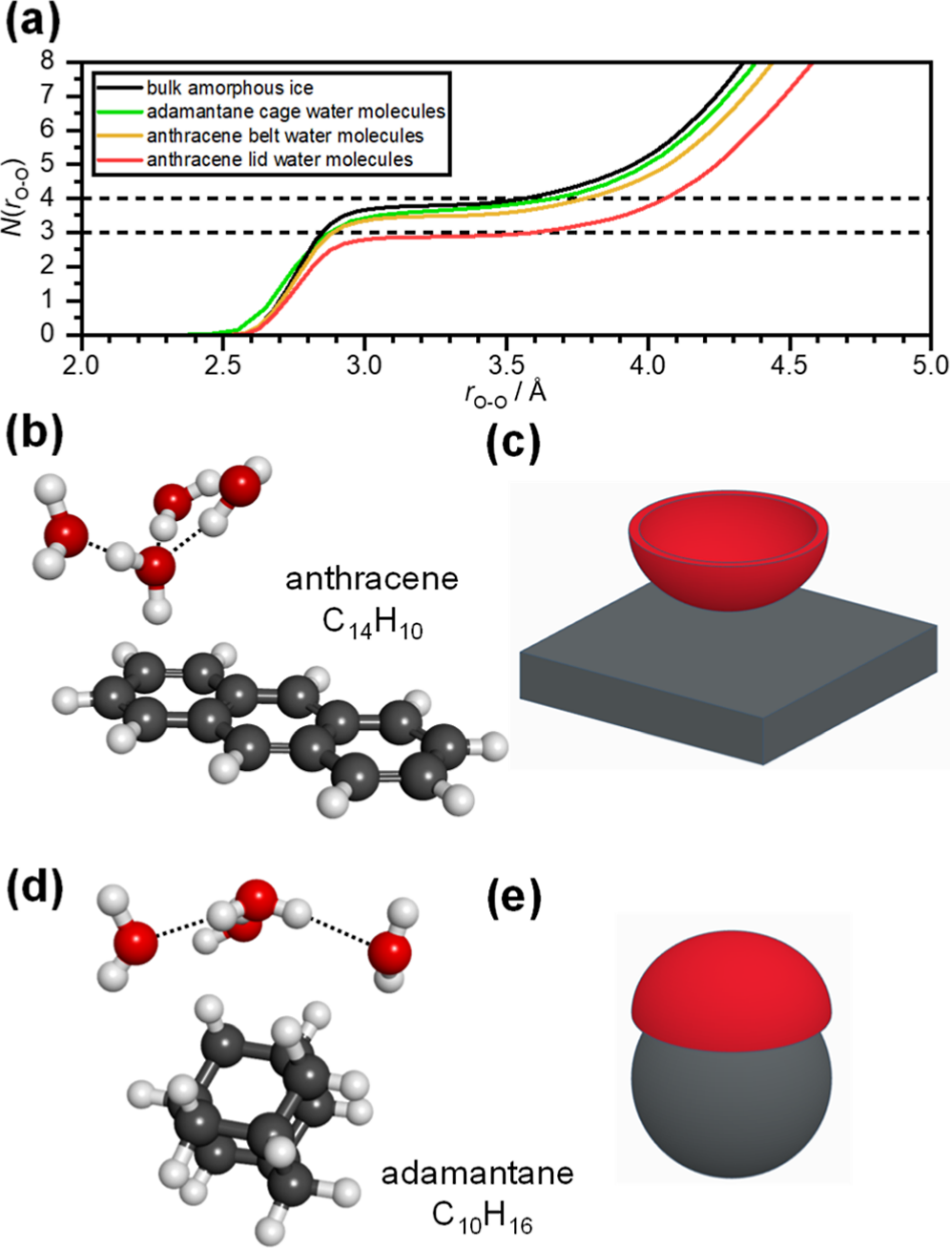
Hydrogen-bonding geometries of the water molecules in the first hydration shell of anthracene. (a) Running oxygen–oxygen atom coordination numbers, *N*(*r*_O–O_), of water molecules in bulk amorphous ice (black), cage water molecules in the first hydration shell of adamantane (green), and lid (red) and belt water molecules (orange) in the first hydration shell of anthracene. (b) Snapshot structure of a lid water molecule in the first hydration shell of anthracene and its hydrogen-bonded neighbors and (c) simplified sketch of the local water geometry. (d) Snapshot structure of a cage water molecule and its hydrogen-bonded neighbors in the first hydration shell of adamantane (C_10_H_16_) and (e) simplified sketch of the local water geometry.^47^ Carbon, oxygen and hydrogen atoms are shown as black, red and white spheres, and hydrogen bonds as black dotted lines.

In contrast to the hydrophobic hydration of anthracene, the water molecules in the first hydration shell of the smaller and nearly spherical adamantane molecule form four hydrogen bonds, similar to the cage structures in clathrate hydrates.^47^ This is evidenced by the plateau in *N*(*r*_O–O_) close to 4 in Figure 4a. The corresponding standard ‘cup’ geometry is shown in the snapshot structure of Figure 4d and depicted schematically in Figure 4e. For comparison, Figure 4a also includes *N*(*r*_O–O_) for bulk amorphous ice, which, as expected, also plateaus at 4. Interestingly, the belt water molecules around anthracene display a plateau in *N*(*r*_O–O_) at around 3.6, suggesting a partial coexistence of the conventional cup and the inverted cup hydration motifs.

The inverted cup hydration motif found for hydrated anthracene may represent a structural marker of hydration at length scales beyond the hydrophobic crossover at least at the low temperatures used in our study. Compared to a fully hydrogen-bonded water molecule with four hydrogen bonds, the inverted cup geometry still allows three hydrogen bonds and enables a fourth, albeit weaker, noncovalent interaction, with the hydrophobe. From an enthalpic point of view, the inverted cup geometry is therefore second best following the standard local bonding environment with four hydrogen bonds. Geometrically speaking, the transition from a standard cup to inverted cup geometry is akin an umbrella being turned inside out by the wind. Consequently, the remaining three hydrogen bonds all point away from the hydrophobe. This effect can then move neighboring hydrogen-bonded water molecules further away from the hydrophobe leading to dewetting phenomena as observed by us at the central ring of anthracene. It is worth noting that similar effects at extended hydrophobic surfaces have also been identified in computational simulations.^43,67^ The dewetting effect observed here is highly local but may represent the onset of the more extended dewetting phenomena predicted for larger hydrophobic surfaces.^3,4,22^

## Conclusions

We have established our low-temperature approach as a method for studying the hydrophobic hydration of large, poorly water-soluble hydrophobic molecules. The low-temperature approach obviously represents a limitation but reflects the fact that higher temperature solutions of large hydrophobic molecules would be very unstable. In this sense, the structural motifs obtained in our studies represent low-temperature limits which should nevertheless serve as meaningful structural “ground states” for more disordered and dynamic hydration structures of large hydrophobes at higher temperatures. In this sense, our low-temperature approach appears to be well suited for probing the enthalpic contributions to hydrophobic hydration. How our structures evolve with increasing temperature toward room temperature is an interesting question that could be addressed by future computational studies, particularly since the experimentally accessible temperature window is relatively small, as illustrated in Figure S2.

Another question is whether our low-temperature samples represent the equilibrium structures. In this context it is worth noting that the as-deposited adamantane/water mixtures at 82 K showed a very similar structure of the first hydration shell compared to after thermally annealing above the glass transition temperature at 140 K.^47^ This suggests that the very local structure at the interface between water and the hydrophobic species is well-equilibrated even just after the deposition. Establishing a well-equilibrated long-range structure is of course expected to be difficult at low temperatures. In fact, we can state with certainty that this is not realized in our samples since this would involve the aggregation or "precipitation" of the hydrophobic species. Such aggregation processes have only been observed for low-temperature C_60_/water mixtures upon increasing the C_60_ content.^46^

Future work should extend our methodology to more structurally complex hydrophobes, particularly those featuring concave hydrophobic surface elements, to explore dewetting phenomena in greater detail. Since the (low-temperature) hydrophobic crossover requires the breaking of water–water hydrogen bonds, the occurrence of noncovalent interactions between the water molecules and the hydrophobic solutes are inevitable. If the strength of the noncovalent interactions has an impact on the length scale of the hydrophobic crossover will be interesting to explore.^41^ In the context of polycyclic aromatic hydrocarbons (PAHs), larger and more complex analogues should be examined to investigate the interplay between molecular size and shape, and the preferred locations of O–H···π bonding.

Furthermore, the present work provides a valuable benchmark for future computational studies and offers a starting point for investigating the pressure dependence of hydrophobic hydration. Moreover, given that PAHs and H_2_O have been found to coexist in cometary materials,^68^ further studies on these mixed systems will enhance our understanding of their spectroscopic signatures, photochemical behavior and water desorption properties. Further insights into the interactions between PAHs and water are also important for atmospheric processes, where PAHs are ubiquitous pollutants as a consequence of incomplete combustion or pyrolysis of coal, oil, wood and petroleum products.^69^

## Supplementary Material



## Notes

Additional X-ray diffraction data (Figure S1 and S2), comparison of experimental and *EPSR* total pair distribution functions (Figure S3), *EPSR* intermolecular pair distribution functions (Figure S4), analysis of the structure of the belt water molecules (Figure S5), neutron weighting factors (Table S1) and details of *EPSR* seed potentials (Table S2) (PDF)
