# Supplementary material for: Structure of Water in the First Hydration Shell of the Highly Hydrophobic Anthracene
Source: J Phys Chem A. 2026 Aug 3;130(32):6356–66. doi: 10.1021/acs.jpca.6c02910 (PMC13479608; doi:10.1021/acs.jpca.6c02910)
Supplement: Supplementary file 1 [file jp6c02910_si_001.pdf]

Supporting Information

Structure of Water in the First Hydration Shell of the Highly Hydrophobic Anthracene

Gustavo A. Madrigal,[1,2] Christos Vasilopanagos,[3] Thomas F. Headen,[4] Alexander Rosu-Finsen,[3] Bharvi Chikani,[3] Sukhpreet K. Talewar,[3] Luis Carlos Pardo,[1,2] Christoph G. Salzmann[3*]

[1] Barcelona Research Center in Multiscale Science and Engineering, Universitat Politècnica de Catalunya, 08019 Barcelona, Spain.

[2] Department of Physics, Universitat Politècnica de Catalunya, 08019 Barcelona, Spain.

[3] Department of Chemistry, University College London, 20 Gordon Street, London WC1H 0AJ, United Kingdom.

[4] ISIS Pulsed Neutron and Muon Source, STFC Rutherford Appleton Laboratory, Harwell Campus, Harwell OX11 0QX, United Kingdom.

Corresponding Author

* c.salzmann@ucl.ac.uk

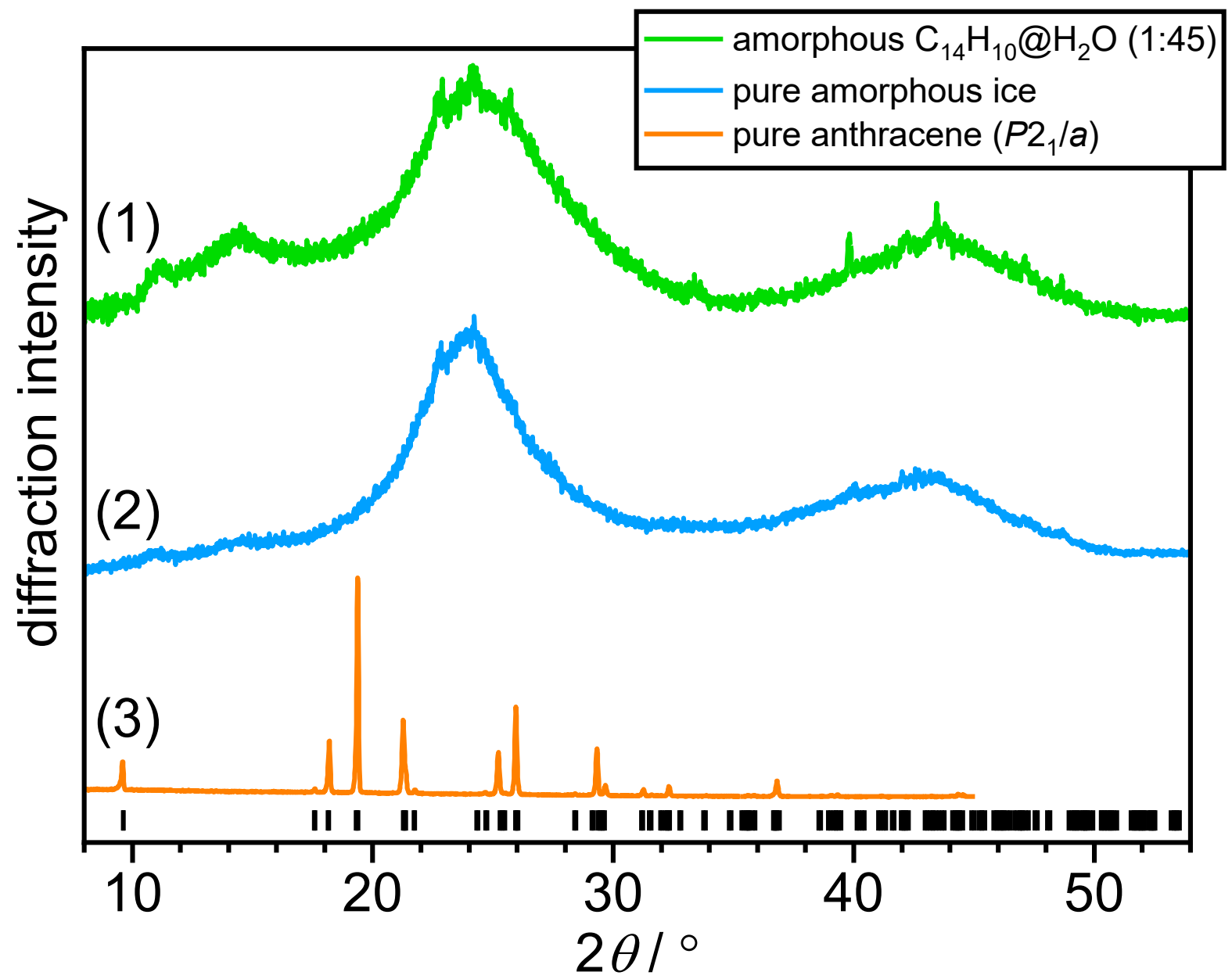

Figure S1. X-ray powder diffraction patterns (λ = 1.540598 Å) of (1) an amorphous mixture of anthracene and H_2O (1:45 molar ratio), (2) pure amorphous ice and (3) pure crystalline anthracene. The first two patterns were recorded at 95 K and the third one at room temperature. The intensities of the diffraction patterns were normalized with respect to the most intense feature, and the diffraction patterns are shifted vertically for clarity. The tick marks indicate the expected positions of the Bragg peaks anthracene.[1]

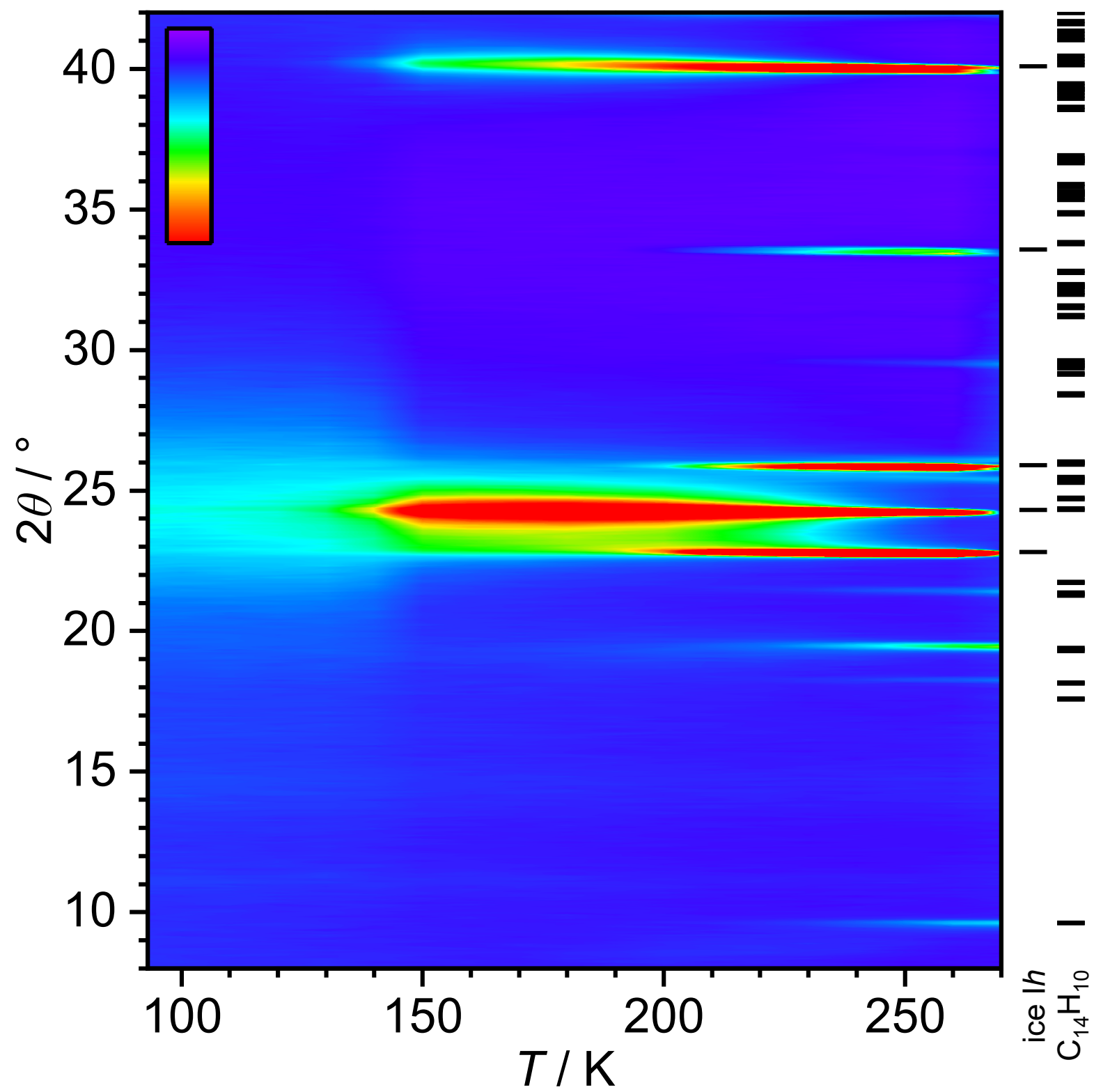

Figure S2. X-ray powder diffraction data (λ = 1.540598 Å) upon heating amorphous $C_{14}H_{10}@H_2O$ (1:45) from 95 to 270 K. Crystallization to stacking-disordered ice I (ice I*sd*)[2] is observed at ~140 K which transforms to hexagonal ice Ih at ~190 K. Anthracene crystallizes slowly beginning at ~190 K. The color scalebar reflects the intensities of the diffraction data. The tickmarks on the right-hand side indicate the expected positions of Bragg peaks of ice I*h*[3] and anthracene ($P2_1$/a).[1]

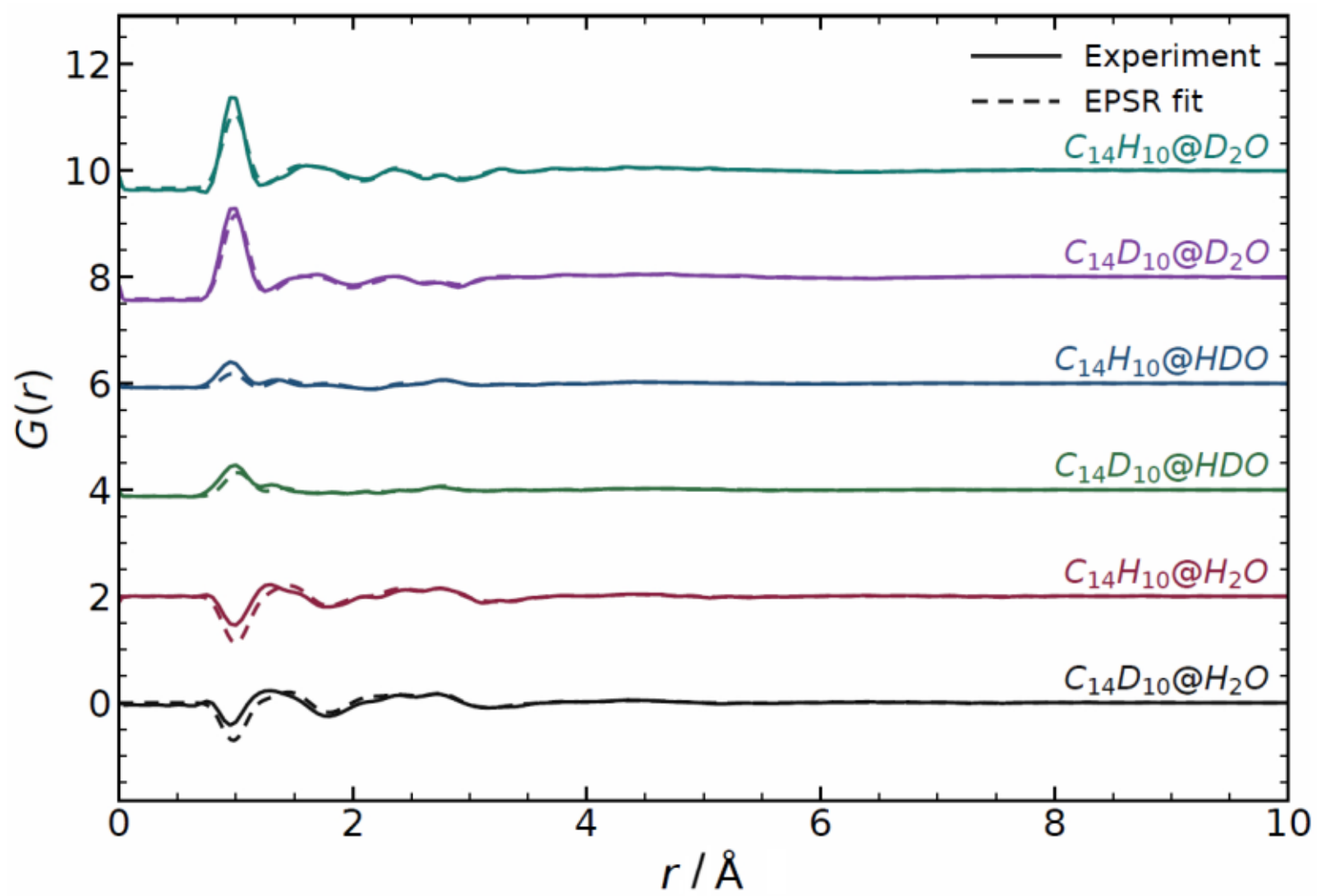

Figure S3. Comparison of the total pair distribution functions, $G(r)$, obtained from the Fourier transform of the experimental structure factors, $F(Q)$, (solid lines) and from the *EPSR* structural models (dashed lines).

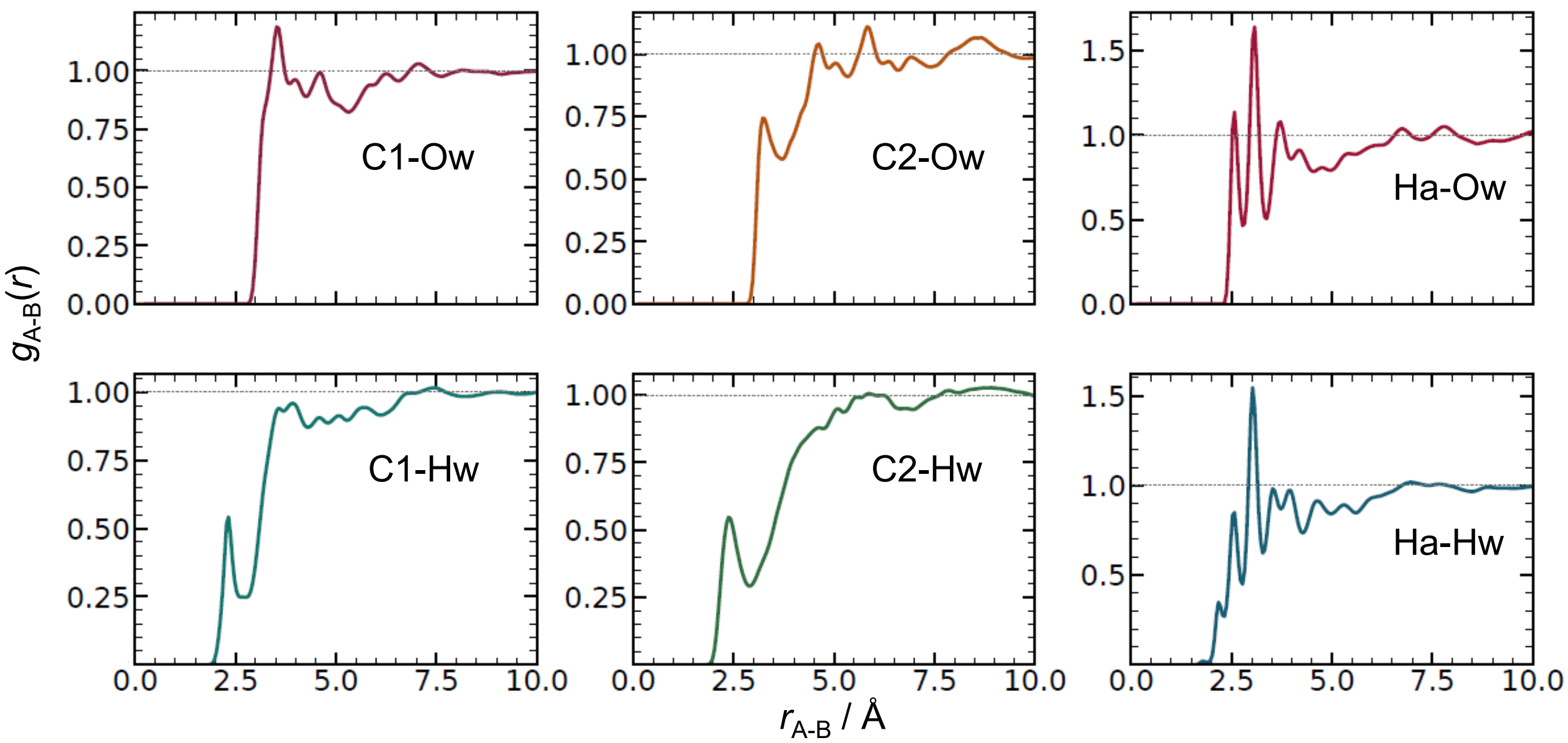

Figure S4. Intermolecular pair distribution functions, $g_{A\text{-}B}(r)$, obtained from the *EPSR* simulation for the various atoms pairs including the secondary (C1) and tertiary carbon atoms (C2) of anthracene, the hydrogen atoms of anthracene (Ha) and water (Hw) as well as the oxygen atoms of water (Ow).

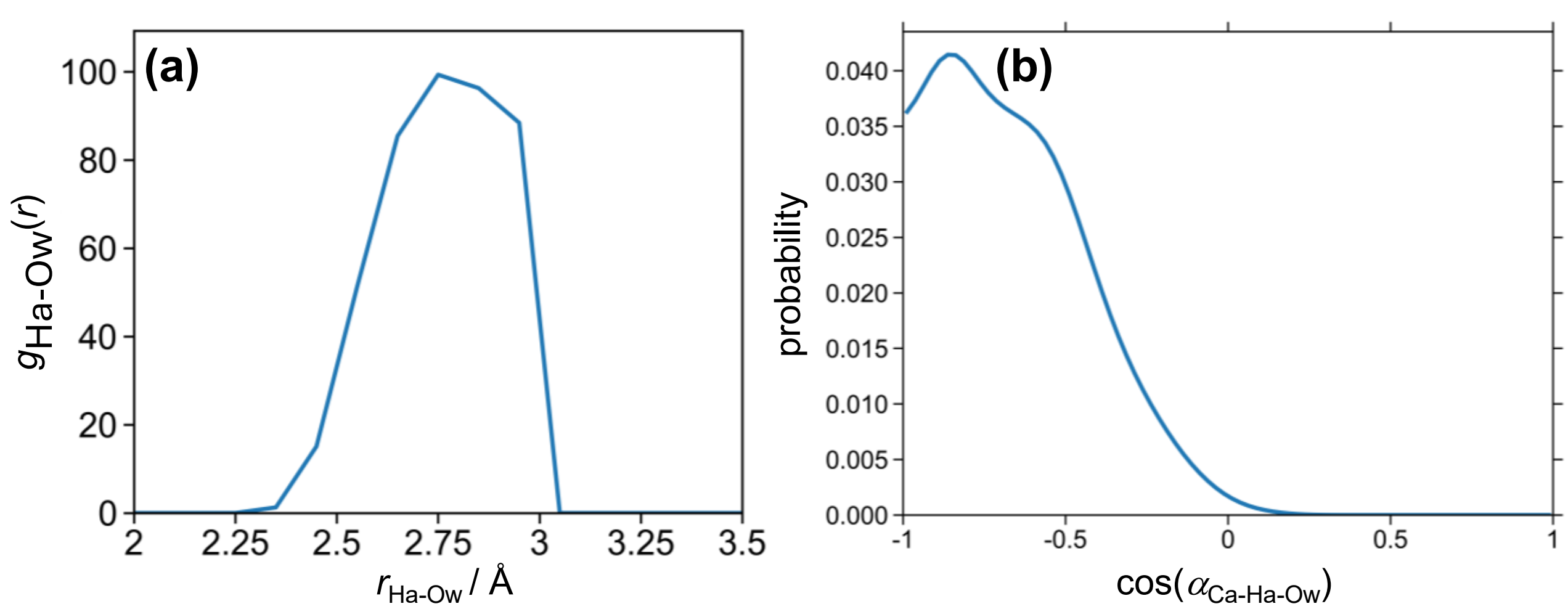

Figure S5. Structure of the belt-molecule hydration shell. (a) Pair distribution function from the hydrogen atoms of anthracene (Ha) to the oxygen atoms of water (Ow). (b) Analysis of the angles spanned by the carbon atoms of anthracene (Ca), the hydrogen atoms of anthracene and the oxygen atoms of water. A Ha-Ow cut off distance of 3.0 Å was used for this analysis.

Table S1. Weighting factors, $w_{A\text{–}B}$, indicating the relative contributions of the ten partial structure factors, $S_{A\text{–}B}(Q)$s, related to the atoms of type A and B to the total structure factors, $F(Q)$s. The hydrogen atoms of anthracene and water are labelled as Ha and Hw, respectively.

$w_{A\text{–}B}$	$C_{14}H_{10}$@H_2O	$C_{14}H_{10}$@HDO	$C_{14}H_{10}$@D_2O	$C_{14}D_{10}$@H_2O	$C_{14}D_{10}$@HDO	$C_{14}D_{10}$@D_2O
$w_{C\text{–}C}$ / fm^2	0.3424	0.3424	0.3424	0.3424	0.3424	0.3424
$w_{C\text{–}Ha}$ / fm^2	-0.1376	-0.1376	-0.1376	0.2455	0.2455	0.2455
$w_{C\text{–}O}$ / fm^2	0.9611	0.9611	0.9611	0.9611	0.9611	0.9611
$w_{C\text{–}Hw}$ / fm^2	-1.2385	0.4856	2.2097	-1.2385	0.4856	2.2097
$w_{Ha\text{–}Ha}$ / fm^2	0.0553	0.0553	0.0553	0.1760	0.1760	0.1760
$w_{Ha\text{–}O}$ / fm^2	-0.3862	-0.3862	-0.3862	0.6891	0.6891	0.6891
$w_{Ha\text{–}Hw}$ / fm^2	0.4977	-0.1951	-0.8880	-0.8880	0.3482	1.5843
$w_{O\text{–}O}$ / fm^2	2.6973	2.6973	2.6973	2.6973	2.6973	2.6973
$w_{O\text{–}Hw}$ / fm^2	-3.4759	1.3628	6.2016	-3.4759	1.3628	6.2016
$w_{Hw\text{–}Hw}$ / fm^2	4.4792	0.6886	14.2585	4.4792	0.6886	14.2585

Table S2. Lennard-Jones parameters, σ and ε, and partial charges, q, used for the seed potentials of the *EPSR* simulation. The secondary and tertiary carbon atoms of anthracene are labelled C1 and C2, and the hydrogen atoms of anthracene and water Ha and Hw.

	C1	C2	Ha	O	Hw
σ / Å	3.55	3.5	2.42	3.1655	0
ε / kJ mol^{-1}	0.29288	0.33472	0.12552	0.6503	0
q / e	0	0	0	-0.82	0.41

References

(1) Mason, R. The Crystallography of Anthracene at 95 K and 290 K. *Acta Cryst.* **1964**, *17*, 547-555.

(2) Malkin, T. L.; Murray, B. J.; Salzmann, C. G.; Molinero, V.; Pickering, S. J.; Whale, T. F. Stacking Disorder in Ice I. *Phys. Chem. Chem. Phys.* **2015**, *17*, 60-76.

(3) Röttger, K.; Endriss, A.; Ihringer, J.; Doyle, S.; Kuhs, W. F. Lattice Constants and Thermal Expansion of H_2o and D_2o Ice Ih between 10 and 265 K. *Acta Cryst.* **1994**, *B50*, 644-648.
